# A Cerebral Origin for Retinal Degeneration in the Visual Form of Alzheimer's Disease?

**DOI:** 10.1111/ejn.70319

**Published:** 2025-11-12

**Authors:** Tristan Jurkiewicz, Elsa Lehingue, Maïté Formaglio, Dominique Kuzdzal, Sarah Verrecchia, Laure Pisella, Caroline Froment Tilikete

**Affiliations:** ^1^ Centre de Recherche en Neurosciences de Lyon (CRNL), INSERM U1028, CNRS UMR5292 Université Claude Bernard Lyon 1 Bron France; ^2^ Service de Neurocognition et de Neuro‐Ophtalmologie Hospices Civils de Lyon Bron France

**Keywords:** optical coherence tomography, posterior cortical atrophy, retinal nerve fibre layer

## Abstract

Alzheimer's disease (ad) manifests commonly as an amnestic syndrome (tad), but also as a rarer focal type, such as posterior cortical atrophy (PCA‐AD), which primarily impairs visuospatial functions. In addition to the brain atrophy, retinal degeneration has been demonstrated, associated with the accumulation of Ab and Tau protein in this tissue, which shares a common origin with the brain. Additionally, retrograde trans‐synaptic degeneration from the brain could affect the retina. We hypothesized that such dying‐back phenomenon would be more important in PCA‐ad than in tad and that this would be reflected on specific optical coherence tomography (OCT) measures. Twenty‐nine AD patients were categorized into 15 typical and 14 PCA forms. Complaints and symptoms were evaluated using a specific screening battery developed to detect PCA (Q‐ACP questionnaire, neuropsychological parietal and non‐parietal scales). Neuroimaging was performed to determine brain atrophy and its lateralization. OCT imaging allowed measuring the volumes of the macular ganglion cell layer (GCL) and the retinal nerve fibre layer (RNFL) of the optic nerve. While the global RNFL thickness and GCL volume were not statistically different, PCA‐ad patients showed more thinning than tAD in the inferior temporal (IT) sector in both eyes. Moreover, the amount of thinning in this sector was correlated with the score on the Q‐ACP questionnaire and on the neuropsychological parietal scales. We propose that the thinning in the IT sector reflects the retrograde damage to the magnocellular pathway, which constitutes a major feed of the dorsal visual stream primarily damaged in PCA.

## Graphical abstract

We compared retinal involvement in typical Alzheimer's disease (tad) and posterior cortical atrophy (PCA‐AD). Although global retinal measures did not differ significantly, we found greater thinning in the inferior temporal (IT) sector in PCA‐ad patients. This thinning was correlated with visuospatial impairments assessed by the Q‐ACP questionnaire and parietal neuropsychological scales. We propose that this sector‐specific thinning reflects enhanced retrograde trans‐synaptic degeneration along the magnocellular pathway, which would correspond to the symptoms of PCA‐ad patients.

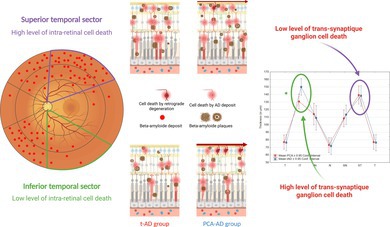

**Abbreviations**
ad
: Alzheimer's disease
AMD: age‐related macular degeneration
BEP: Battery of Praxia evaluation
BREF: Frontal Efficiency Rapid Battery
CSF: cerebrospinal fluid
FDR: false discovery rate
FE: far eccentricity retina
G: global
GCL: ganglion cell layer
HLH: homonymous lateral hemianopia
IADL: questionnaire used to assess patient autonomy in daily life
IN: inferonasal
IT: inferotemporal
ME: medium eccentricity retina
MMSE: Mini Mental State Evaluation
N: nasal sectors of the RNFL
OCT: optical coherence tomography
ONH: optic nerve head
PCA: posterior cortical atrophy
PCA‐AD: posterior cortical atrophy form of Alzheimer's disease
PLPS: post‐lumbar puncture syndrome
PP: posterior pole
Ptau: hyperphosphorylated tau protein
Q‐ACP: questionnaire about the visuo‐spatial impacts on daily activities of PCA
Q‐ACP: questionnaire of daily life difficulties specific to PCA
RNFL: retinal nerve fibre layer
SN: superonasal
ST: superotemporal
T: temporal
tad
: typical amnestic syndrome of Alzheimer's disease
VF: visual field
VOSP: Visual Object and Space Perception Battery

## 1 Introduction

The recognized pathological hallmarks of Alzheimer's disease (ad) patients are characterized by amyloid β‐protein plaques and neurofibrillary tangles composed of hyperphosphorylated tau protein (Ptau) deposition in the brain (Perrin et al. 2009; Selkoe 2008; Avila et al. 2016) contributing to inflammation and neuronal loss (De Strooper and Karran 2016).

The retina shares many structural and functional features with the brain, due to a common origin in the embryonic diencephalon. These include populations of neurons and glial cells that secrete proteins linked to the amyloid cascade (Morin et al. 1993; Mirzaei et al. 2020).

Pathological signs specific to ad have recently been identified in the human retina (Gaire et al. 2024) with accumulations contributing to direct neuroretina degeneration and a decrease in the retinal nerve fibre layer (RNFL). However, retinal degeneration could also be explained by an additive dying‐back phenomenon: retinal ganglion cell axons degenerate retrogradely from cerebral synaptic connections (Zhang et al. 2022; Alladi et al. 2007).

RNFL impairment in ad (review in La Morgia et al. 2017) has been most studied in its typical form (tad, around 85% of ad presentations), which begins with hippocampal memory loss. ad can also present as focal cortical dysfunction such as posterior cortical atrophy (PCA‐ad) estimated at 5% prevalence (Snowden et al. 2007; Koedam et al. 2010). In PCA‐AD, memory remains relatively spared, as well as language and executive functions; predominant symptoms concern visual function and are associated with cortical atrophy that predominates in the posterior part of the hemispheres (Benson et al. 1988). Given this localization of lesions in regions processing visual information, we hypothesized that, if there is retinal degeneration resulting from a dying‐back phenomenon, it could be more extensive in PCA‐ad than in tad patients. One previous study found no difference in RNFL thickness between tad and PCA‐AD patients (den Haan et al. 2019). Here, we aimed to further investigate this hypothesis by employing a more sensitive RNFL measurement involving more sectors. More specifically, we explored different predictions based on the current consensus classification of PCA‐ad (Crutch et al. 2017), which highlights symptoms reflecting the selective early degeneration of parietal, followed by occipital cortices (Kas et al. 2011).

The first prediction is that the bilateral posterior parietal atrophy could be the best candidate to originate a potential dying‐back because parietal damage is the earliest in the PCA‐ad disease progression (Kas et al. 2011). Accordingly, in terms of symptoms, PCA‐AD is characterized by early and prominent disturbance of visuospatial and visuoperceptual functions pertaining to the Balint's syndrome, and also of other cognitive functions pertaining to Gerstmann's syndrome (Crutch et al. 2017). The bilateral dorsal visual pathways are characterized by spatial representations of the environment without cortical magnification of central vision, therefore with larger representation of peripheral vision (Galletti et al. 1999; Pitzalis et al. 2015), with some maps specialized to detect a visual event across the entire visual field (Corbetta and Shulman 2002). Peripheral visual field narrowing frequently observed in PCA‐ad patients in both eyes could reflect this bilateral posterior parietal damage. To test for this first prediction, we therefore compared RNFL measures between tad and PCA‐ad groups, in both eyes, without taking the lateralization of the damage into account.

However, this hypothesis of a lack of effect of brain lateralization on OCT measures remained to be checked by comparing RNFL measures between tAD and PCA‐AD groups and taking the hemispheric lateralization visible by neuroimaging into account. To make this verification, we selected the patients with hemispheric lateralization visible by neuroimaging (bilateral parietal damage is often asymmetric) and coded the eyes as ipsilateral or contralateral to the predominant side of cerebral atrophy.

The alternative prediction that can be made is that the occipital atrophy is the best candidate to originate a potential dying‐back because it is more purely visual. The occipital cortex, even if it may be damaged later (Kas et al. 2011), may be more proximal to the retina. This occipital atrophy is often predominant in one hemisphere, as reflected by the high prevalence of left or right homonymous lateral hemianopia (HLH) in PCA‐ad (Olds et al. 2020; Formaglio et al. 2009; Pellegrini et al. 2017). A dying‐back linked to such lateralized homonymous visual field defects would lead to an increased thinning in a different retinal sector in each eye: For example, left hemianopia in the context of right primary occipital damage would have retrogradely affected the temporal sector of the left eye and the nasal sector of the right eye. In order to test for this second prediction, we therefore selected patients with hemianopia and coded the eyes as ipsilateral or contralateral.

## 2 Method

### 2.1 Recruitment Protocol

The study was undertaken within ad patients newly diagnosed in the Memory Resource and Research Center (CMRR), as a typical or PCA form of ad between 2018 and 2024. The protocol was approved by the institutional review board of the Hospices Civils de Lyon, classified as category 3 non‐interventional. All included patients were informed of the study and provided their consent. This study was carried out according to the MR004 criteria of the Commission Nationale de l'Informatique et des Libertés (CNIL; n°19‐387).

Inclusion criteria were a progressive course of symptoms suggesting either a typical amnestic form (tad) or a PCA form (PCA‐ad) of ad. This further involved the presence of cerebral atrophy on MRI and a biologically confirmed diagnosis of ad on CSF analysis (McKhann et al. 2011). A Mini Mental State Evaluation (MMSE) score of 16 or above was also required for being included in the present study (Table 1). Cerebral atrophy was classified as predominantly left (L), right (R) or symmetrical (S) by a neuroradiologist, blind to the clinical presentation, based on visual assessment of whole‐brain MRI and further PET scan examination. The CSF analysis was based on the level of Amyloid (≤ 550 ng/L), Tau (≥ 400 ng/L) and Ptau (≥ 60 ng/L). If there was any doubt about the diagnosis of ad on CSF analysis, the Abeta 42/40 ratio (≥ 0.055) was used to confirm the ad diagnosis (Formaglio et al. 2011; Seguin et al. 2011).

**TABLE 1:** Neuropsychological screening battery to compute the ‘right parietal’, the ‘left parietal’ and the ‘non‐parietal’ scales. BEP: Battery of Praxia evaluation (Peigneux and Van der Linden 2000), VOSP: Visual Object and Space Perception Battery (Warrington and James 1991).

‘Right Parietal’ scale		‘Left Parietal’ scale		‘Non‐parietal’ scale	
Items	Rating	Items	Rating	Items	Rating
Cancellation test	1 pt	Left/right disorientation on patient's body parts	1 pt	Spatial orientation (on which town and level of the building are we?)	1 pt for the town, and 1 pt for the level
Description of a visual scene	1 pt if global apprehension, 0 pt by details	Left/right disorientation on experimenter's body parts	1 pt		
Navon test	2 pts if spontaneous, 1 pt if assisted	Left/right disorientation on letter drawing (GPCDJ)	1 pt	Temporal orientation (which month and day are we?)	1 pt for the month and, 1 pt for the day
		Digital agnosia on patient's body	1 pt		
Dot counting of VOSP	2 pts if correct without manual aid, 1 pt otherwise	Digital agnosia on experimenter's body	1 pt	Immediate recall of three words of MMSE	1 pt
		Put on calculation on paper (1023 + 978)	1 pt	Delayed recall of three words of MMSE	1 pt
Copy of a geometrical sequence	1 pt	Result of the calculation (1023 + 978)	1 pt	Sentence repetition of MMSE	1 pt
Figure copy of MMSE	1 pt	Mental calculation of MMSE	1 pt	Instruction understanding of MMSE	1 pt
Spontaneous daisy drawing	1 pt	Unimanual praxia of BEP	1 pt	Similitude test of BREF	1 pt
Reading of a very short text	1 pt	Bimanual praxia of BEP	1 pt	Verbal fluency of BREF	1 pt
Simple denomination of line drawn objects (BARD scale)	1 pt	Praxia on imitation of BEP	1 pt	Go/no go test of BREF	1 pt
Object prehension	1 pt if object detected, 1 pt if object manually captured	Spontaneous writing of patient's name	1 pt	Working memory span	1 pt
		Writing of a sentence verbally given by the experimenter	1 pt	Working memory span (reversed)	1 pt
10 items	/13 pts	13 items	/13 pts	11 items	/13 pts

Exclusion criteria were pathologies, other than AD, that could have an impact on optic nerve or retina (e.g., glaucoma, age‐related macular degeneration, diabetic retinopathy, …). These were detected during a systematic ophthalmology consultation, with an ocular examination, refraction evaluation, visual acuity measures on the Monoyer scale and optical coherence tomography (OCT).

### 2.2 Data Collection

**FIGURE 1:**
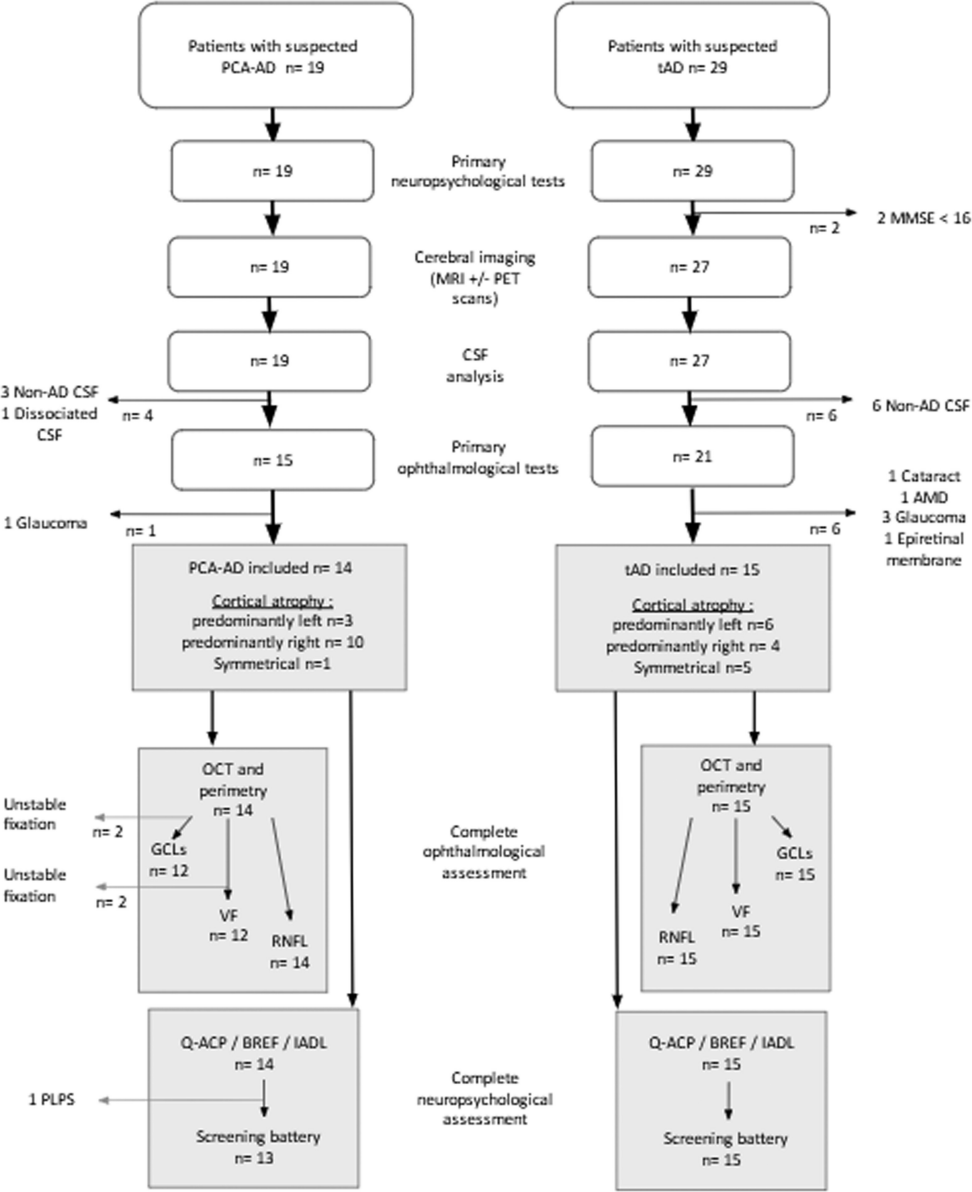
Flow chart. The white sections represent the patient recruitment process. The grey sections represent the further hemispheric lateralization, ophthalmological and neuropsychological examinations carried out on the recruited patients*.* The general and specific questionnaires about the impacts on daily activities (IadL and Q‐ACP, respectively) were filled out by all recruited patients. Missing data occurred because of unstable fixation preventing visual field (VF) perimetry or measurement of the volume of the ganglion cell layer (GCL), or because of the patients' condition preventing the full neuropsychological assessment (PLPS = post‐lumbar puncture syndrome). AMD = age‐related macular degeneration, CSF = cerebrospinal fluid.

Further neuro‐ophthalmological and cognitive assessments were undertaken on the recruited population (Figure 1) including visual field perimetry (MonCv3, Metrovision, France), general and specific questionnaires about the impacts on daily activities (questionnaire used to assess patient autonomy [IADL] and French questionnaire of daily life difficulties specific to PCA [Q‐ACP, Croisile and Mollion 2011, a translation in English can be accessed online and uploaded at https://doi.org/10.1080/01658107.2025.2507407], respectively), as well as detailed OCT and neuropsychological measures described below.

#### 2.2.1 Detailed OCT Measures

OCT measurements were performed using the Spectralis OCT (Heidelberg Engineering Inc., Heidelberg, Germany; software ver.1.10.12.0). A real‐time eye‐tracking system measured eye movements and provided feedback to the scanning mechanism to stabilize the retinal position on the B‐scan. A posterior pole scan (30° × 25°, 61 sections at 120‐μm intervals, 768 A‐scans) and consecutive peripapillary retina scans RNFL‐N (diameter of 12°, 1536 A‐scans) were acquired without pupil dilation. The parameters were automatically measured using the respective analysis algorithms. The volume of the ganglion cell layer (GCL) was extracted from the results of the posterior pole scan. The average thickness of global (G), superotemporal (ST), inferotemporal (IT), temporal (T), superonasal (SN), inferonasal (IN) and nasal (N) RNFL was extracted from the peripapillary retina scans.

#### 2.2.2 Detailed Neuropsychological Measures

A full neuropsychological assessment was undertaken, starting with the general standardized scale of the Frontal Efficiency Rapid Battery (BREF) and followed by a screening battery that was elaborated in order to complete the neurocognitive evaluation and compute the ‘right parietal’, the ‘left parietal’ and the ‘non‐parietal’ scales (Table 1). The ‘Right Parietal’ scale was inspired by Balint's syndrome usually observed after right and/or bilateral parietal damage. It thus included tests of optic ataxia, simultanagnosia, neglect and constructive apraxia. The ‘Left Parietal’ scale was inspired by Gerstmann's syndrome usually observed after left inferior parietal damage. It thus included tests of agraphia/dysgraphia, acalculia/dyscalculia, left–right confusion and finger agnosia. The ‘non‐parietal’ scale evaluated spatial and temporal orientation as well as immediate and working memory, language and executive functions.

### 2.3 Statistical Analyses

The present study evaluated the different predictions using the detailed OCT and cognitive measures, in the whole population of patients first, then in a selected population of patients presenting hemispheric lateralization and, finally, in a selected population of patients with hemianopia.

The statistics were performed with Statistica software (StatSoft Inc., Tulsa, Oklahoma; ver.14.0.0.15). Student's *t* tests were performed on age, global RNFL thickness, ganglion cell volume, global cognitive functions (MMSE, BREF) and quality of life (IadL) to ensure that the groups of PCA‐ad and tAD patients were comparable.

The thicknesses of the different sectors of the papillary OCT were analysed using repeated measures ANOVAs, with Group (PCA/tAD), Sectors (T/ST/SN/N/IN/IT) and Eye as factors. For the first analysis, performed in the whole population, the Eye factor was classified as Right/Left. LSD pairwise comparisons were used to test the planned direct comparisons of the least squares means and further understand the significant interaction effects. Corrections for multiple comparisons were applied using the Benjamini‐Hochberg method, with a false discovery rate (FDR) threshold of 5%.

Correlations between RNFL thickness or ganglion cell volume and neuropsychological test scores (MMSE, IADL, BREF, Q‐ACP, left parietal scale, right parietal scale and non‐parietal scales) were performed using Pearson correlations merging the data of the two eyes of the patients. These correlations were performed both in the whole population and in the population of lateralized patients.

For the second and third analyses, performed only in selected populations, the Eye factor was classified as Ipsilateral/Contralateral to the cerebral lateralization.

In order to test whether the lateralization of the lesion determined from neuroimaging was representative of the cognitive impairment of the patient indicated by the scores on the right and left parietal scales, we carried out a factorial ANOVA on these scores with Group of lateralized patients (PCA/tAD) and lesion lateralization (right/left) as factors. The same analysis was also run on the non‐parietal scale.

## 3 Results

### 3.1 Description of the Whole Population

Fourteen PCA patients and 15 tAD patients were included (at the end of the recruitment process, see flow chart and Table 2) for further neuro‐ophthalmological and neuropsychological assessment. The two groups were matched on age (66.86 ± 6.65 for PCA and 66.20 ± 7.34 years old for tad; *t*(27) = 0.25; *p* = 0.80), disease duration (3.79 ± 2.01 years for PCA and 3.87 ± 2.14 years for tad; *t*(27) = −0.10; *p* = 0.91), CSF protein levels (there were no significant differences between groups for the level of amyloid [632.29 ± 242.04 for PCA and 638.87 ± 240.61 for tad; *t*(21) = −0.073; *p* = 0.94], Tau [685.93 ± 408.41 for PCA and 571.07 ± 261.58 for tAD; *t*(21) = 0.91; *p* = 0.37] and Ptau [98.29 ± 46.78 for PCA and 92.13 ± 41.68 for tAD; *t*(21) = 0.37; *p* = 0.71]), global neuropsychological scales (MMSE [22.00 ± 2.91 for PCA and 23.75 ± 2.77 for tAD; *t*(27) = −1.51; *p* = 0.14] and BREF [12.64 ± 3.20 for PCA and 14.07 ± 2.91 for tAD; *t*(27) = −1.25; *p* = 0.22]) and global autonomy (IADL questionnaire [2.79 ± 1.12 for PCA and 2.67 ± 1.50 for tAD; *t*(27) = 0.24; *p* = 0.81]).

**TABLE 2:** Descriptive data from patients included in the study.

Group	Patient ID	Disease duration (years)	Lateralization	Age	Sex	Visual field RE	Visual field LE	MMSE	Q‐ACP	IADL	BREF	Right parietal scale	Left parietal scale	Non‐parietal scale	Amyloid level (ng/L)	Tau level (ng/L)	Ptau level (ng/L)	Ab 40/42 ratio
PCA	1	4	L	63	F	Peripheral narrowing	No impairment	23	9	1	11	6	2	11	668	376	70	
	2	3	L	73	F	Right HLH	Right HLH	19	10	3	12	6	5	9	559	436	68	
	3	3	L	60	M	Right HLH	Right HLH	23	19	3	11				567	1493	213	0.036
	4	5	R	74	F	Peripheral narrowing	No impairment	24	15	3	15	6	4	10	525	406	76	
	5	3	R	60	F	Left HLH	Left HLH	27	19	2	18	4	8	11	452	477	74	
	6	8	R	68	M	Not feasible	Not feasible	19	22	3	12	0	7	7	686	599	78	
	7	2	R	68	M	Left HLH	Left HLH	23	16	1	15	1	8	9	566	717	99	
	8	7	R	72	F	Left HLH	Left HLH	23	21	4	16	1	7	24	465	533	91	
	9	3	R	77	M	Peripheral narrowing	No impairment	21	23	4	11	0	7	8	451	460	65	
	10	5	R	71	F	Left HLH	Left HLH	16	29	1	12	3	5	4	695	518	84	0.059
	11	5	R	64	F	Left HLH	Left HLH	24	10	3	17	8	10	12	530	423	75	0.051
	12	2	R	67	F	Not feasible	Not feasible	22	11	4	7	6	9	9	571	570	65	
	13	1	R	52	F	Left HLH	Left LHL	25	18	3	12	4	8	11	1417	1680	188	
	14	2	S	67	F	Peripheral narrowing	Peripheral narrowing	19	17	4	8	6	3	6	700	915	130	
tAD	15	2	L	58	M	No impairment	No impairment	25	1	1	16	13	12	10	542	484	71	
	16	4	L	62	F	No impairment	No impairment	25	2	1	15	12	9	7	567	551	83	
	17	4,5	L	74	F	Peripheral narrowing	Peripheral narrowing	23	2	4	14	9	10	10	654	539	87	0.049
	18	9	L	56	F	Extended blind spot	Extended blind spot	27	0	4	16	10	11	9	1335	628	78	0.073
	19	2	L	77	F	No impairment	No impairment	25	0	4	14	11	11	11	529	814	105	0.037
	20	4	L	64	F	No impairment	No impairment	22	0	4	15	13	12	7	626	1027	108	
	21	4	R	68	F	Extended blind spot	No impairment	23	0	1	16	11	11	9	649	772	96	0.062
	22	2	R	58	F	Extended blind spot	No impairment	23	6	1	11	9	9	7	484	401	78	
	23	3	R	68	M	No impairment	Left inferior scotoma	27	1	4	18	12	11	11	749	316	66	0.074
	24	2	R	64	M	Peripheral narrowing	Peripheral narrowing	23	0	4	7	9	10	6	434	487	84	0.061
	25	1,5	S	64	F	No impairment	No impairment	21	1	1	15	13	11	8	401	168	34	0.073
	26	2	S	74	F	No impairment	Extended blind spot	27	1	1	16	13	10	9	515	506	76	
	27	6	S	69	F	No impairment	No impairment	24	3	4	15	10	8	10	699	1110	204	0.039
	28	6	S	58	M	No impairment	No impairment	23	0	4	14	12	12	9	437	317	53	
	29	6	S	79	M	No impairment	No impairment	16	2	2	9	10	7	4	962	446	159	

*Note:* The Ab 40/42 ratio (last column) was calculated in cases where CSF indices were unclear about the diagnosis of AD in order to confirm it.

Abbreviations: F = female, HLH = homonymous lateralized hemianopia, LE = left eye, M = male, RE = right eye.

Visual field evaluation (Table 2) showed that eight PCA‐ad patients presented (left or right) hemianopia contralesional to the side of their predominant cortical damage determined on neuroimaging and four PCA‐AD patients presented peripheral narrowing and three among them nevertheless displayed some lateralization in their cerebral damage; two PCA‐AD patients with right hemispheric lateralization could not be examined because of unstable ocular fixation. In the tAD group, two patients presented peripheral narrowing, one patient displayed a left inferior scotoma on the eye contralesional to the side of the predominant cortical damage determined on neuroimaging, four presented extended blind spots and eight had no visual field impairment at all.

### 3.2 Analyses Performed on the Whole Patients' Population

#### 3.2.1 Neuropsychological Analysis

The scores did not differ between the two groups for the non‐parietal scale of the neuropsychological screening battery (8.77 ± 2.31 for PCA and 8.47 ± 1.96 for tad; *t*(26) = 0.37; *p* = 0.71). On the other hand, there was a significant deterioration in the PCA‐AD group on the right (3.92 ± 2.69 for PCA and 11.13 ± 1.55 for tAD; *t*(26) = −8.83; *p* < 0.001) and the left (6.38 ± 2.40 for PCA and 10.27 ± 1.49 for tAD; *t*(26) = −5.22; *p* < 0.001) parietal scales of the screening battery, as well as on the Q‐ACP questionnaire (17.07 ± 5.77 for PCA and 1.27 ± 1.62 for tAD; *t*(27) = 10.20; *p* < 0.001).

#### 3.2.2 OCT Analysis

The GCL volume was not statistically different between the two groups of patients (1.06 ± 0.11 mm^3^ for PCA and 1.04 ± 0.07 mm^3^; *t*(27) = 0.76; *p* = 0.46).

The global RNFL thickness was not statistically different between the two groups of patients (100.57 ± 7.89 μm for PCA and 99.40 ± 5.31 μm for tAD; *t*(27) = 0.47; *p* = 0.64).

**FIGURE 2:**
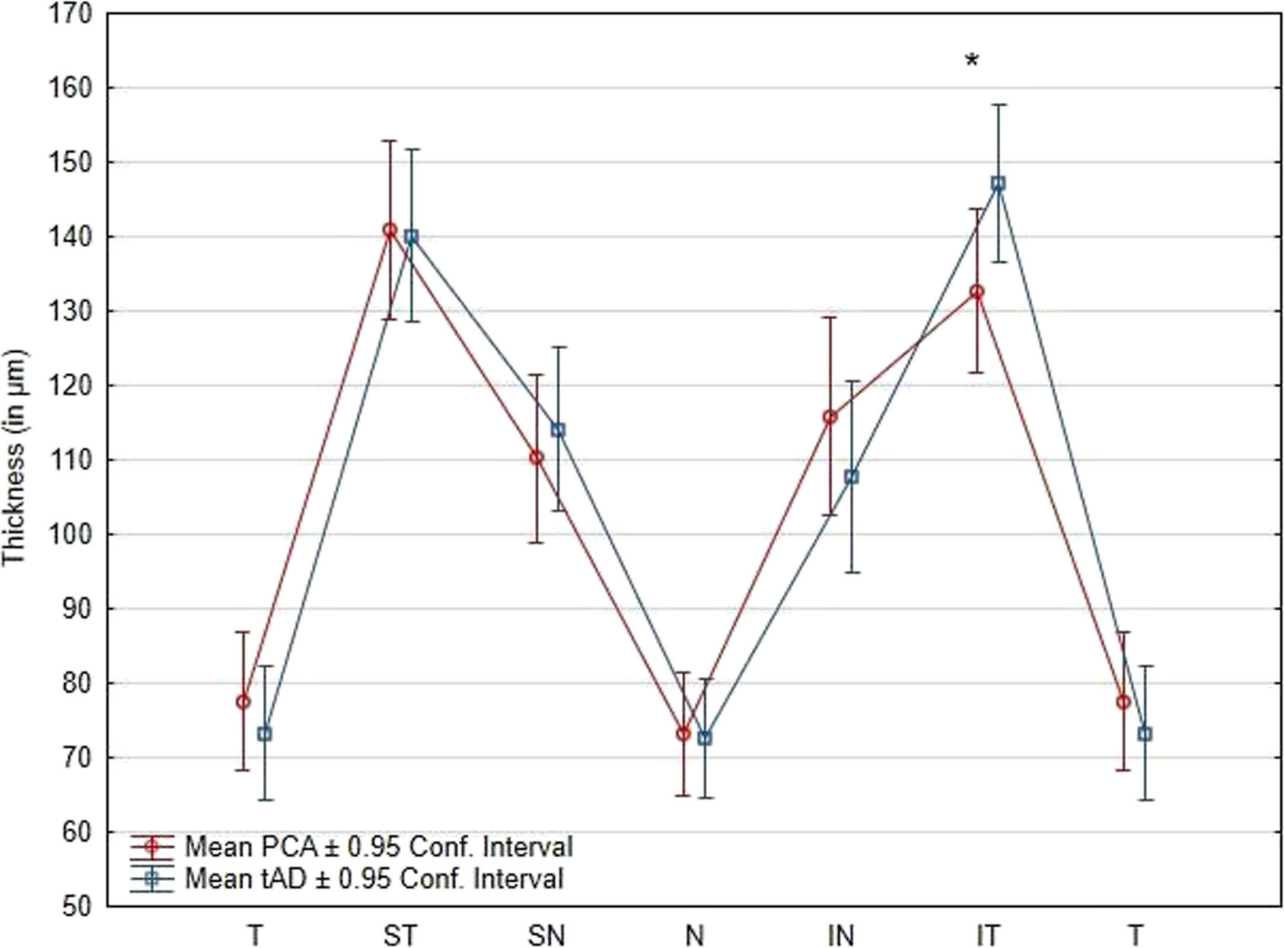
Significant interaction of Group and Sectors on RNFL thickness. IN = inferonasal, IT = inferotemporal, N = nasal, SN = superonasal, ST = superotemporal, T = temporal. Error bars correspond to 95% Confidence Intervals. *Significant pairwise comparison between groups after FDR correction.

The repeated measures ANOVA assessing the RNFL thickness difference by Group (tAD/PCA‐AD), Eye (right/left) and Sectors (T/ST/SN/N/IN/IT) revealed a main effect of Eye (*F*(1, 27) = 5.9, *p* = 0.022, *η*_2_ = 0.18) corresponding to a mean thickness larger in the right eye. There was also a main effect of the OCT Sector (*F*(5, 135) = 145.24, *p* < 0.001, *η*_2_ = 0.84), the ST and IT sectors displaying thickness significantly higher than all other sectors. There was no main effect of group (*F*(1, 27) = 0.1, *p* = 0.78, *η*_2_ = 0.0037), but the two‐way interaction between Sector and Group was significant (*F*(5, 135) = 2.6, *p* = 0.029, *η*_2_ = 0.09); post hoc analyses showed a significant decrease of the thickness in the IT sector in the PCA group compared to the tAD group (*F*(27) = 7.63; *p* = 0.01) (Figure 2). All other interactions were not significant (all *F*s < 2.2; all *p*s > 0.062).

#### 3.2.3 Correlation Analysis Between Cognitive and OCT Measures

**FIGURE 3:**
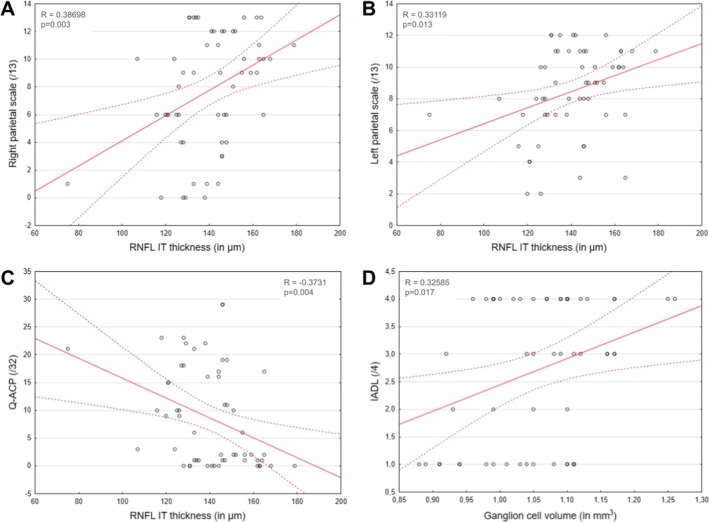
Correlations between OCT and cognitive measures. Significant correlation of RNFL IT thickness with (A) right parietal scale, (B) left parietal scale of the screening battery and (C) Q‐ACP. (D) Significant correlation between GCL volume and IadL score.

RNFL IT thickness showed a significant positive correlation with the right (*R* = 0.39; *p* = 0.003) and the left (*R* = 0.33; *p* = 0.013) parietal scales (the thinner the IT sector, the lower the parietal scores) and a significant negative correlation with the Q‐ACP (*R* = −0.38; *p* = 0.004, the thinner the RNFL IT sector, the higher the score at the Q‐ACP questionnaire developed to enumerate the PCA symptoms). The correlation analysis carried out on GCL volume showed a significant positive correlation with the IadL questionnaire (*R* = 0.32; *p* = 0.017, the higher the GCL volume, the higher the autonomy score) (Figure 3, Table 3).

**TABLE 3:** Correlation coefficients and *p*‐values between cognitive and neuro‐ophthalmological measures.

		Ganglion cell volume	Inferior temporal RNFL
MMSE	*R*	0.15	0.09
	*p*‐value	0.278	0.521
Q‐ACP	*R*	0.02	−0.36
	*p*‐value	0.889	0.007^a^
IADL	*R*	0.32	−0.24
	*p*‐value	0.017^a^	0.078
BREF	*R*	0.07	−0.01
	*p*‐value	0.623	0.952
Right parietal scale	*R*	0.07	0.39
	*p*‐value	0.603	0.003^a^
Left parietal scale	*R*	0.17	0.33
	*p*‐value	0.211	0.013^a^
Non‐parietal scale	*R*	0.27	−0.15
	*p*‐value	0.047	0.282

^a^ Significant after FDR correction.

### 3.3 Analyses Performed on the Selected Population of Patients With Lateralized Brain Damage

We selected patients with lateralized cerebral damage (13 PCA and 10 tAD patients) observable from neuroimaging (labelled R for right and L for left on Table 1). In the PCA‐AD group, cortical atrophy was predominantly right in 10 patients and left in 3. In the tAD group, cerebral atrophy was predominantly right in 4 patients and left in 6. The two groups were matched on age (*p* = 0.51) and disease duration (*p* = 0.84), CSF protein levels (amyloid [*p* = 0.78], Tau [*p* = 0.65], Ptau [*p* = 0.52]), global neuropsychological function and level of autonomy (MMSE [*p* = 0.060], BREF [*p* = 0.36], IADL questionnaire [*p* = 0.85]). There was a significant deterioration in the scores of the PCA‐AD group on the Q‐ACP questionnaire (17.08 ± 6.01 for PCA and 1.20 ± 1.87 for tAD; *t*(21) = 8.03; *p* < 0.001).

#### 3.3.1 Analysis of Cognitive Impairment as a Function of Lateralization Determined by Neuroimaging

**FIGURE 4:**
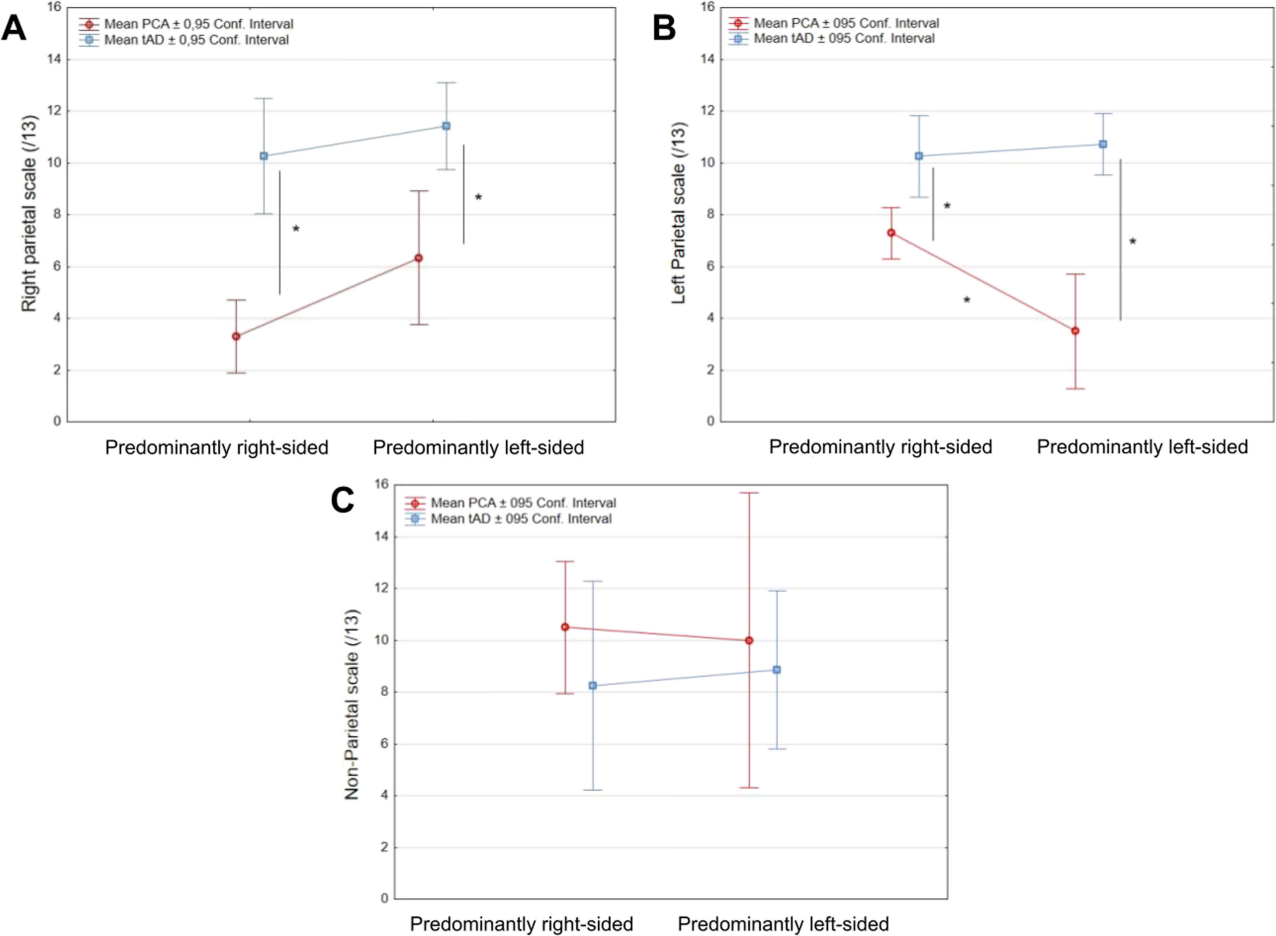
Interactions between patients' lateralization and group (tad/PCA‐ad) for performance in neuropsychological screening battery. (A) Non‐significant interaction between Lateralization and Group for the right parietal scale. There was nevertheless a significant main effect of the Lateralization and of the Group. (B) Significant interaction between Lateralization and Group for the left parietal scale. (C) Non‐significant interaction between Lateralization and Group for the non‐parietal scale. *Post hoc tests significant after FDR correction.

Factorial ANOVA was performed separately for the three scales of the neuropsychological screening battery with the factors of Group (tAD/PCA) and Lateralization (Left/Right). The results are illustrated in Figure 4.

For the right parietal scale, a main effect of Group (*F*(1, 18) = 29.62, *p* < 0.001, *η*_2_ = 0.67) was observed with lower scores in the PCA group than in tad. This difference between groups was also significant separately for patients with predominantly left or right lesions (*F*(18) = 10.61; *p* = 0.0044 and *F*(18) = 27.55; *p* < 0.001, respectively). There was also a main effect of Lateralization (*F*(1, 18) = 4.71, *p* = 0.042, *η*_2_ = 0.20), patients with right hemispheric lateralization displaying lower scores. The interaction between the Group and the Lateralization did not reach statistical significance (*F*(1, 18) = 0.51, *p* = 0.48, *η*_2_ = 0.14).

For the left parietal scale, a main effect of Group (*F*(1, 18) = 44.38, *p* < 0.001, *η*_2_ = 0.71) was also observed with lower scores in the PCA group than in tAD, as well as an interaction between the Group and the Lateralization (*F*(1, 18) = 8.06, *p* = 0.011, *η*_2_ = 0.59). Post hoc tests showed a significant decrease in the score of the left parietal scale for PCA compared to the tAD group, for both patients with left and right predominant damage (*F*(18) = 34.42; *p* < 0.001 and *F*(18) = 8.52; *p* = 0.0092, respectively). Within the PCA group, the score of the left parietal scale was also significantly lower in predominantly left lesions than in predominantly right lesions (*F*(18) = 10.27; *p* = 0.0049).

For the non‐parietal scale, the means were inferior in the tAD group compared to PCA but this did not reach statistical significance (all *F*s(1, 18) < 0.78, *p*s > 0.39), as already observed for the standardized measures of general cognitive abilities.

#### 3.3.2 Analysis of OCT Measures as a Function of Lateralization Determined by Neuroimaging

GCL volume was not statistically different between the two groups of patients (1.05 ± 0.11 mm^3^ for PCA and 1.03 ± 0.07 mm^3^ for tAD; *t*(21) = 0.46; *p* = 0.65). Global RNFL thickness was not statistically different between the two groups of patients (99.69 ± 7.46 μm for PCA and 99.65 ± 5.15 μm for tAD; *t*(21) = 0.015; *p* = 0.99).

**FIGURE 5:**
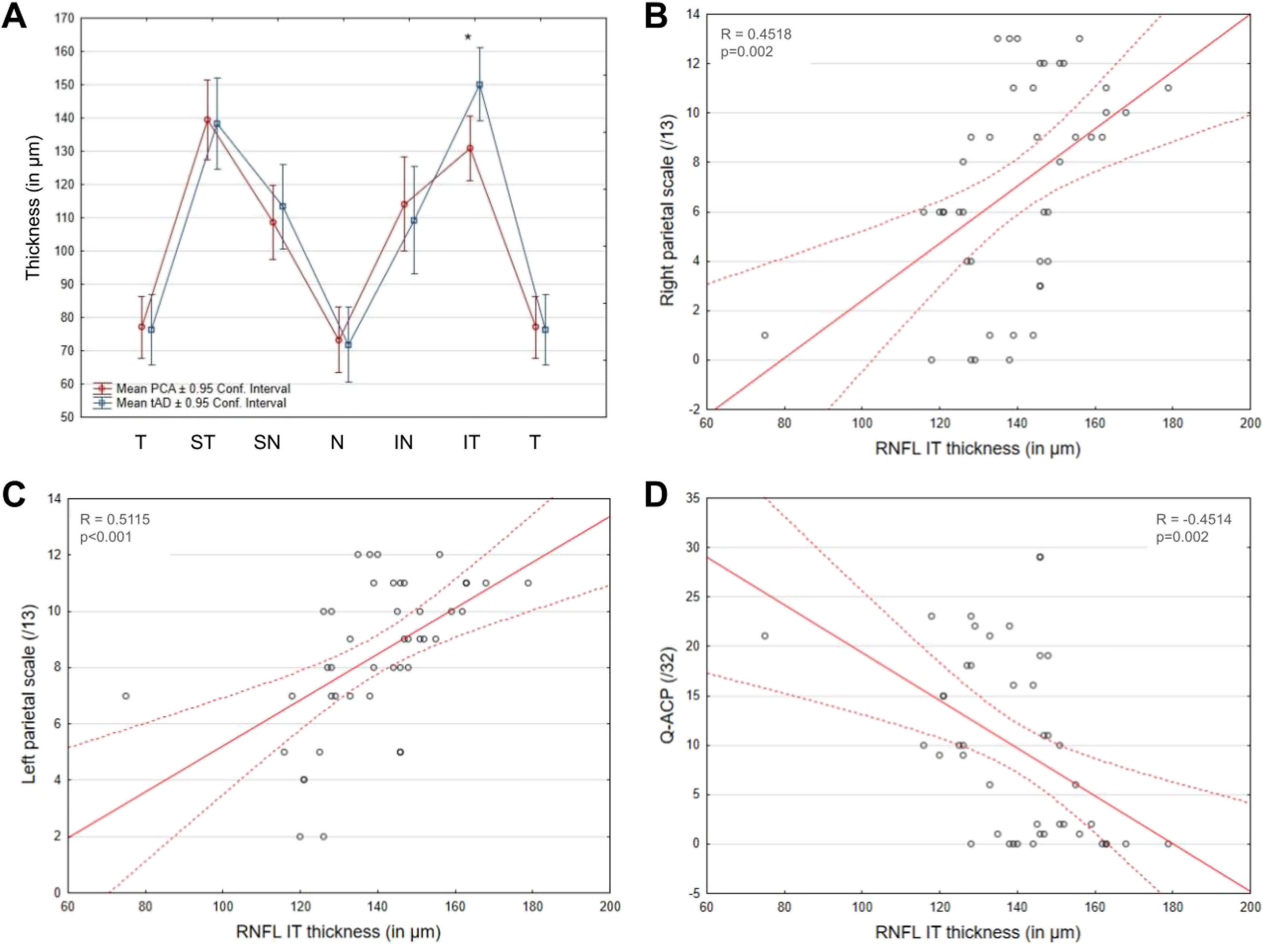
Summary of significant results of the analyses of OCT measures performed in the selected population of lateralized patients. (A) Significant interaction of Group and Sectors on RNFL thickness. IN = inferonasal, IT = inferotemporal, N = nasal, SN = superonasal, ST = superotemporal, T = temporal. Error bars correspond to 95% confidence intervals. *Significant after FDR correction. (B, C and D) Illustrations of the significant correlations between RNFL IT thickness and cognitive measures in lateralized PCA‐AD and tAD patients. (B) Right parietal scale, (C) left parietal scale and (D) Q‐ACP.

The repeated measures ANOVA assessing OCT thickness difference with Group, Eye (ipsilateral/contralateral to hemispheric lateralization) and Sector revealed a main effect of Eye (*F*(1, 21) = 6.8, *p* = 0.016, *η*_2_ = 0.24) corresponding to a mean thickness significantly thinner in the eye contralateral compared to the eye ipsilateral to the predominant cortical damage. There was also a main effect of the OCT Sectors (*F*(5, 105) = 116.48, *p* < 0.001, *η*_2_ = 0.85), the ST and IT sectors displaying thickness significantly higher than all other sectors. There was no main effect of group (*F*(1, 21) = 0.6, *p* = 0.41, *η*_2_ = 0.028), but the two‐way interaction between Sector and Group was significant (*F*(5, 105) = 2.48, *p* = 0.037, *η*_2_ = 0.11); post hoc analyses showed a significant decrease of the thickness in the IT sector in the PCA group compared to the tad group (*F*(21) = 12.52; *p* = 0.0019) (Figure 5A). All interactions involving the Eye were not significant (all *F*s < 2.5; all *p*s > 0.13).

#### 3.3.3 Correlation Analysis Between Cognitive and OCT Measures

A significant correlation of RNFL thickness of the IT sector was observed with the right (*R* = 0.45; *p* = 0.002) and the left (*R* = 0.51; *p* < 0.001) parietal scales of the neuropsychological screening battery and with the Q‐ACP questionnaire (*R* = −0.45; *p* = 0.002) (Figure 5B/C/D). Correlations with all other cognitive measures were not significant (all *R*s < 0.23 and all *p*s > 0.132). The same correlation analysis was carried out between GCL volume and cognitive measures with none reaching significance after FDR correction (all *R*s < 0.33 and all *p*s > 0.031).

#### 3.3.4 Analyses Performed on the Selected Population of Patients With Hemianopia

**FIGURE 6:**
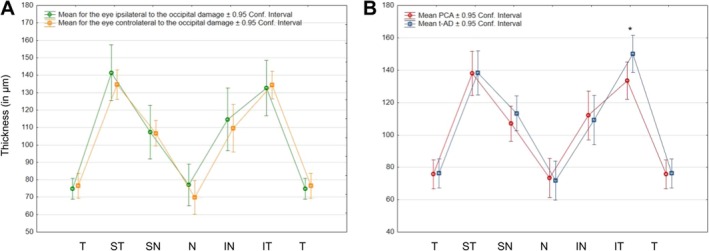
Summary of significant results of the analyses of OCT measures performed in the population of PCA patients with hemianopia. (A) Non‐significant interaction of Eye and Sectors on RNFL thickness. (B) Non‐significant interaction of Group and Sectors on RNFL thickness. IN = inferonasal, IT = inferotemporal, N = nasal, SN = superonasal, ST = superotemporal, T = temporal. Error bars correspond to 95% confidence intervals. *Significant after FDR correction.

As the eight patients presenting hemianopia pertained to the group of PCA‐ad patients, the repeated measures ANOVA performed on the thicknesses of the different sectors of the papillary OCT included only two factors: the OCT Sector (T/ST/SN/N/IN/IT) and the Eye (Ipsilateral/contralateral to the occipital damage). Repeated measures ANOVA showed a main effect of the Sector (*F*(5, 35) = 29.33; *p* < 0.001, *η*_2_ = 0.81) and a main effect of the Eye (*F*(1, 7) = 8.70; *p* = 0.021, *η*_2_ = 0.037) corresponding to a mean thickness that was significantly thinner in the eye contralateral to the occipital damage. The interaction was not significant (*F*(5, 35) = 0.49; *p* = 0.78, *η*_2_ = 0.065) and no post hoc comparisons between the eyes at each sector reached significance (All *F*s < 2.29; all *p*s > 0.17) (Figure 6A).

Another repeated measures ANOVA contrasted these eight PCA patients with hemianopia and the 10 tad patients with lateralized brain damage. There was no main effect of Group (*F*(1, 18) = 1.2; *p* = 0.29, *η*_2_ = 0.063) and no main effect of Eye (*F*(1, 18) = 4.2; *p* = 0.056, *η*_2_ = 0.19). The main effect of Sector (*F*(5, 90) = 116.0; *p* < 0.001, *η*_2_ = 0.87) was found. The Group × Sector interaction did not reach significance (*F*(5, 90) = 1.8; *p* = 0.13), but the post hoc analysis confirmed the significant thinning in PCA patients of the inferior temporal sector (*F*(18) = 9.26; *p* = 0.007), with no other significant difference (all *F*s < 1.52; all *p*s > 0.23) (Figure 6B). The other interactions were not significant (all *F*s < 0.9; all *p*s > 0.36).

## 4 Discussion

Together with the brain atrophy, retinal degeneration has been demonstrated in ad, due to the accumulation of Ab and Tau protein in both tissues, which share a common origin with the brain. Additionally, retrograde trans‐synaptic degeneration from the brain could affect the retina. While studies are interested in using retinal examination as a potential noninvasive diagnosis and tracking of Alzheimer's disease (Gaire et al. 2024), our study focused on the underlying physiopathology of such retinal degeneration. We hypothesized that the dying‐back phenomenon would be earlier or more distinct in PCA‐ad than in tad and that this would be reflected on OCT.

To resume, our results showed that macular ganglion cell volume and global RNFL thickness did not differ between tad and PCA‐ad groups. Only the RNFL sector analysis revealed that PCA‐AD displayed additional thickness reduction in the inferior temporal (IT) sector compared to tAD, independent of the eye and the cerebral lateralization. Moreover, this very specific OCT measure was correlated with the importance of functional deficit reported on the Q‐ACP questionnaire and the left and right parietal scales of the neuropsychological screening battery. As developed below, we put forward that these results are in favour of our first prediction of a dying‐back phenomenon originating from the early bilateral posterior parietal atrophy in the visual form of ad.

The posterior parietal cortex corresponds to the dorsal visual stream specialized in spatial processing and global vision. According to the early bilateral posterior parietal atrophy, the first difficulties in daily activities of PCA‐AD patients reflect Balint's syndrome and may concern reading (simultanagnosia, visual crowding and ocular apraxia), visuomotor performance (optic ataxia, constructional dyspraxia, object and space perception deficit), spatial discrimination and orientation (Hof et al. 1989; Crutch et al. 2017; Yong et al. 2023). Although some spatial maps of this bilateral network represent more of the contralateral visual field, they are oculocentric rather than strictly retinotopic (Colby et al. 1995), which requires afferents that come from both visual hemifields. Moreover, specialized parietal maps in the right hemisphere in humans allow detection of a visual event across the entire visual field (Corbetta and Shulman 2002). The bilateral dorsal visual stream is also characterized by a spatial representation of the environment without cortical magnification of central vision; therefore, it has a large representation of peripheral vision (Galletti et al. 1999; Pitzalis et al. 2015). A dying‐back originating from the posterior parietal cortex could therefore affect global/peripheral vision.

This prediction is in line with the non‐macular pattern of retinal degeneration reported in ad reflected by specific thinning in the inferior and superior sectors of the ON and suggesting preferential degeneration of Magnocellular and Koniocellular ganglion cells (Pablo Piñero et al. 2016; La Morgia et al. 2017; Sartucci et al. 2010, 2020). These two pathways each account for 10% of total ganglion cells (Casagrande 1994; Shapley and Perry 1986), which can explain the small thinning of the RNFL, which remained within the normal range, in ad patients. This contrasts with other neurodegenerative pathologies, such as Parkinson's and Huntington's disease, where the retinal damage is more extensive and preferentially affects the macula and thus the Parvocellular pathway (La Morgia et al. 2017), which accounts for 80% of total ganglion cells (Shapley and Perry 1986).

**FIGURE 7:**
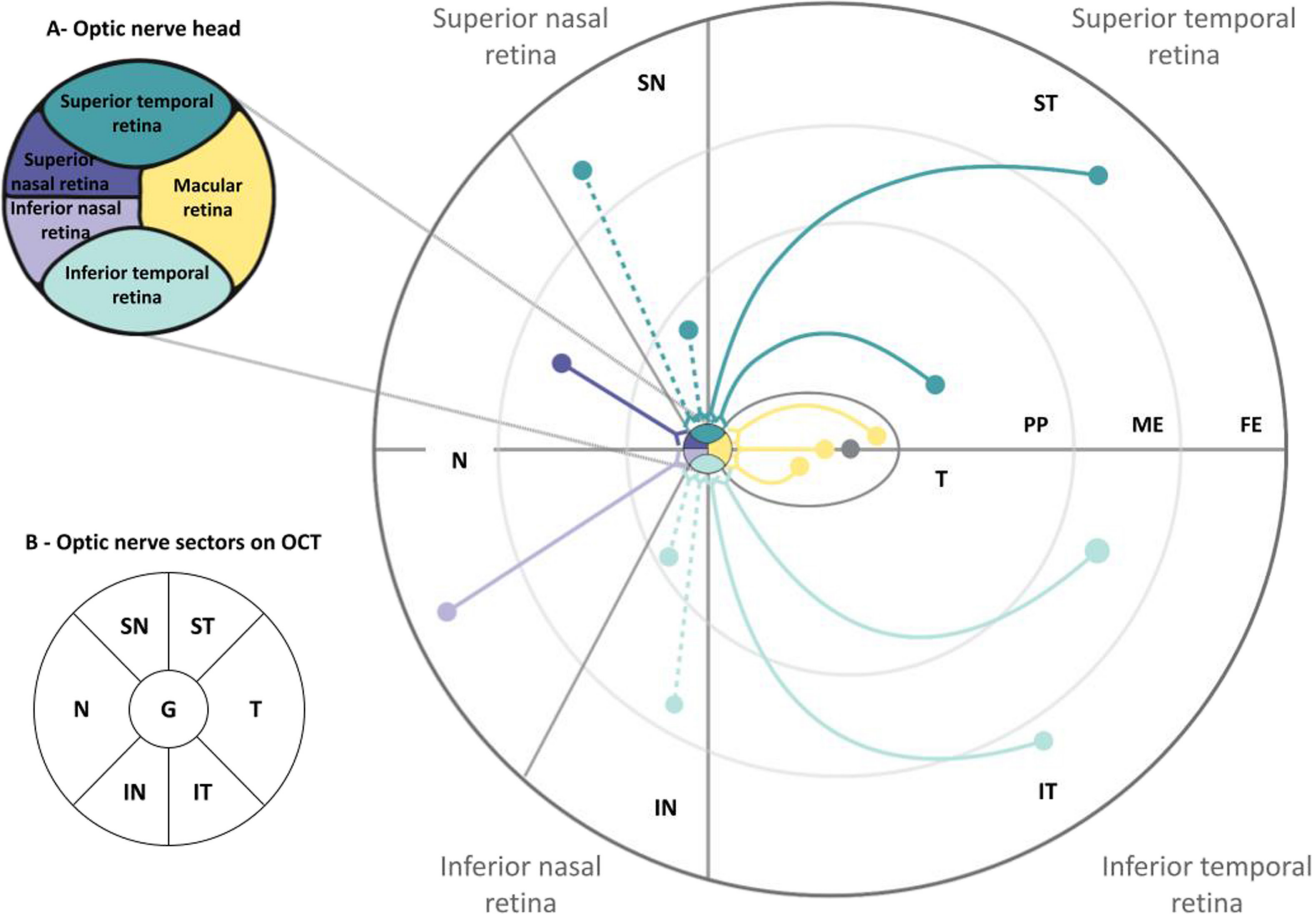
Schematic representation of the course of ganglion cell axons from different regions of the retina to the optic nerve head (based on La Morgia et al. 2017; Pawar et al. 2024; Standring 2020). The macula is represented by the shaded area in the centre of the retina. In relation to the macula, the first circle represents the posterior pole (PP), the second the medium eccentricity (ME) retina and the third the far eccentricity (FE) retina. Axons from nasal ganglion cells (dotted arrows) enter the optic nerve head radially. In contrast, axons emerging from the temporal part of the retina (solid arrows) form curved trajectories to avoid the macular zone (papillomacular bundle). Axons (yellow solid arrows) originating in the macular zone (grey oval) project directly onto the optic nerve head. (A) Distribution of retinal nerve fibres in the optic nerve head (ONH) on the left eye. (B) Schematic representation of the sectors on the RNFL‐N of the left eye in correspondence with the retinal origin of ganglion cells in the ONH. Global (G), superotemporal (ST), inferotemporal (IT), temporal (T), superonasal (SN), inferonasal (IN) and nasal (N). The temporal parts (IT, ST and T) decussate at the level of the optic chiasm, while the nasal parts do not (IN, SN and N).

Though, the significant thinning in PCA‐ad patients compared with tad patients was observed only in the IT sector of the ON. This sector projects onto the ipsilateral visual cortex without decussation at the level of the optic chiasm (Pawar et al. 2024) and receives from the most peripheral retina. Note that the nasal and temporal retinas have physiologically a lower RNFL thickness than other areas, and are particularly well preserved in ad. On the contrary, the superior and inferior retinas, with greater RNFL thickness, are affected especially in the medium and far eccentricity (Figure 7) (Koronyo‐Hamaoui et al. 2020; Koronyo et al. 2017; Mirzaei et al. 2020). A closer look at the inferior retina reveals that its temporal part is more preserved than the nasal part (Koronyo‐Hamaoui et al. 2020). This appears to be the sector of the ON, with a relatively high RNFL thickness, where the difference between the tad and PCA‐ad is significant. The location where the difference was found could therefore be linked to a better sensitivity of measure in this sector, which is the most preserved from direct ad lesions.

We wish to argue that the absence of effect of the predominance of the cerebral atrophy on the RNFL in the analysis involving the patients with lateralized neuroimaging profiles does not contradict our hypothesis of retrograde neurodegeneration from posterior parietal visual areas. Most patients were actually bilaterally damaged. The lateralization of cortical atrophy determined from neuroimaging was only a predominance, as shown by the cognitive deficits of the patients at our screening battery. In fact, the groups of PCA‐AD patients categorized with right or left lateralization both displayed lower scores than tAD patients at both right and left parietal scales (even if the patients categorized with right cerebral damage dominance were more severely impaired at the right than at the left parietal scale and vice versa). Unlike the neuropsychological deficits observed on both parietal scales, the lateralized deficits of the visual field measured in these PCA‐AD patients were almost exclusively contralateral to the cerebral hemisphere categorized as predominantly affected based on neuroimaging. Yet, it should be noted that the eye did not constitute a factor interacting with the group by sector effect, whether it was classified as contralesional/ipsilesional to the predominantly damaged hemisphere (in the second analysis), or whether it was simply included as left/right (in the first analysis). Therefore, despite the most frequent visual field deficits observed in the PCA‐AD group, the present results were not explained by a dying back resulting from occipital damage at the level of the primary visual area leading to hemianopia. Instead, the impairment was observed in the same IT sector of the ON in both eyes which reflects a preferential impairment of the magnocellular and/or koniocellular pathways, whose retinal ganglion cells are homogeneously distributed in the retina outside the fovea. These ganglion cells predominate in the peripheral retina, while the number of other ganglion cell types is very low. Moreover, the retinal ganglion cells of the magnocellular and/or koniocellular pathways are the most extended, in line with their characteristic visual impairment of global vision.

A previous article in the literature compared RNFL in patients between PCA and tad; without finding significant differences (den Haan et al. 2019). An explanation could be that the examinations were carried out with a different OCT. The literature has already highlighted the differences between the two systems. We used the spectralis OCT, which first displayed a greater inter‐operator reproducibility, high repeatability and a lower variation in measurements (SD) (Wolf‐Schnurrbusch et al. 2009; Pierro et al. 2010). Second, the thickness measured was greater due to a different algorithm but also by different limits to calculate the thickness; the Spectralis OCT identifies the external reflective band as the outer border (Wolf‐Schnurrbusch et al. 2009; Pierro et al. 2010; Sachdev et al. 2016). Furthermore, the analyses carried out were different in the previous study, in which only 4 sectors were studied (I/S/T/N). If our results are confirmed and the specific thinning is found only in IT, the averaging of IT and IN could explain the absence of difference in the study of den Haan et al. (2019).

It should be finally noted that our study has some limitations, particularly in regard to the number of patients. This is due to enrolment being conducted as part of standard patient follow‐up in our memory clinic with time‐consuming step‐by‐step inclusion of rare PCA‐AD patients. The low number of patients is also due to the strict requirement to have a diagnosis based on clinical, neuropathological, radiological and neuropsychological assessment for inclusion in the study, which is also, in our opinion, a strength of this study.

## Author Contributions

**Tristan Jurkiewicz:** data curation, formal analysis, investigation, writing – original draft. **Elsa Lehingue:** investigation. **Maïté Formaglio:** investigation, project administration. **Dominique Kuzdzal:** investigation. **Sarah Verrecchia:** investigation. **Laure Pisella:** methodology, supervision, writing – original draft, writing – review and editing. **Caroline Froment Tilikete:** conceptualization, methodology, project administration, supervision, writing – review and editing.

## Conflicts of Interest

The authors declare no conflicts of interest.

## Data Availability

All relevant data supporting the findings of this study are openly available in the Figshare database at DOI: https://doi.org/10.6084/m9.figshare.29493185.
